# Tuberculosis in South Asia: A regional analysis of burden, progress, and future projections using the global burden of disease (1990–2021)

**DOI:** 10.1016/j.jctube.2024.100480

**Published:** 2024-10-09

**Authors:** Vijay Kumar, Mahalaqua Nazli Khatib, Amit Verma, Sorabh Lakhanpal, Suhas Ballal, Sanjay Kumar, Mahakshit Bhat, Aryantika Sharma, M. Ravi Kumar, Aashna Sinha, Abhay M. Gaidhane, Muhammed Shabil, Mahendra Pratap Singh, Sanjit Sah, Kiran Bhopte, Kamal Kundra, Shailesh Kumar Samal

**Affiliations:** a Evidence for Policy and Learning, Global Center for Evidence Synthesis, Chandigarh, India; b Division of Evidence Synthesis, Global Consortium of Public Health and Research, Datta Meghe Institute of Higher Education, Wardha, India; c Department of Medicine, Graphic Era Institute of Medical Sciences, Graphic Era (Deemed to be University), Clement Town, Dehradun, India; d School of Pharmaceutical Sciences, Lovely Professional University, Phagwara, India; e Department of Chemistry and Biochemistry, School of Sciences, JAIN (Deemed to be University), Bangalore, Karnataka, India; f Department of Allied Healthcare and Sciences, Vivekananda Global University, Jaipur, Rajasthan 303012, India; g Department of Medicine, National Institute of Medical Sciences, NIMS University Rajasthan, Jaipur, India; h Chandigarh Pharmacy College, Chandigarh Group of Colleges-Jhanjeri, Mohali 140307, Punjab, India; i Department of Chemistry, Raghu Engineering College, Visakhapatnam, Andhra Pradesh 531162, India; j Uttaranchal Institute of Pharmaceutical Sciences, Division of Research and Innovation, Uttaranchal University, India; k Jawaharlal Nehru Medical College, and Global Health Academy, School of Epidemiology and Public Health, Datta Meghe Institute of Higher Education, Wardha, India; l University Center for Research and Development, Chandigarh University, Mohali 140413 Punjab, India; m Medical Laboratories Techniques Department, AL-Mustaqbal University, 51001 Hillah, Babil, Iraq; n Center for Global Health Research, Saveetha Medical College and Hospital, Saveetha Institute of Medical and Technical Sciences, Saveetha University, Chennai, India; o SR Sanjeevani Hospital, Kalyanpur, Siraha 56517, Nepal; p Department of Paediatrics, Dr. D. Y. Patil Medical College, Hospital and Research Centre, Dr. D. Y. Patil Vidyapeeth, Pune 411018, Maharashtra, India; q Department of Public Health Dentistry, Dr. D.Y. Patil Dental College and Hospital, Dr. D.Y. Patil Vidyapeeth, Pune 411018, Maharashtra, India; r IES Institute of Pharmacy, IES University, Bhopal, Madhya Pradesh 462044, India; s New Delhi Institute of Management, Delhi, India; t Unit of Immunology and Chronic Disease, Institute of Environmental Medicine, Karolinska Institutet, 17177 Stockholm, Sweden

**Keywords:** Tuberculosis, South Asia, Global burden of disease, ARIMA, Joinpoint regression

## Abstract

**Background:**

Tuberculosis (TB) is a major public health issue in South Asia and accounts for a large share of the global TB burden. Despite global efforts to curb TB incidence and mortality, progress in South Asia has been uneven, necessitating focused regional analysis to guide effective interventions. This study aims to analyse the trends in the TB burden in South Asia from 1990 to 2021 and project future TB incidence rates up to 2031.

**Methods:**

This study utilized data from the Global Burden of Disease (GBD) 2021 results to analyse trends in age-standardized incidence (ASIR), prevalence (ASPR), mortality (ASMR), and disability-adjusted life years (DALYs) rates (ASDR) associated with TB in South Asia from 1990 to 2021. Joinpoint regression analysis was employed to identify significant trends, whereas ARIMA models were used to project future TB incidence rates up to 2031.

**Results:**

This study revealed significant declines in the ASIR, ASPR, ASDR, and ASMR related to TB in South Asia over the past three decades. Prominent reductions were found in Bangladesh and Bhutan, whereas India, Pakistan, and Nepal continue to bear the highest TB burdens. The ARIMA model projections indicate a continued decline in TB incidence across the region, although the extent of the decline varies by country, with less favourable trends observed in Nepal and Pakistan. The analysis also highlights tobacco use, high fasting plasma glucose, and high body mass index as significant risk factors contributing to the TB burden.

**Conclusions:**

Substantial progress has been made in reducing the TB burden in South Asia; however, sustained and intensified efforts are needed, particularly in countries with inconsistent progress. These findings emphasize the need for targeted interventions to meet the WHO End TB Strategy (WETS) targets by 2035. Continuous monitoring and adaptive strategies will be crucial in maintaining and accelerating progress toward TB elimination in South Asia.

## 1 Introduction

Tuberculosis (TB) remains a significant global health challenge, particularly in South Asia, where it continues to be a major contributor to morbidity and mortality despite being a preventable and largely curable disease. The region accounts for a major part of the global TB burden, with countries such as India (27 %), Pakistan (5.7 %), and Bangladesh (3.6 %) experiencing some of the highest incidence rates worldwide [1]. TB causes more than 1.35 million deaths annually and was the leading cause of death due to a single infectious agent as recently as 2021 [2]. This enduring burden highlights the ongoing challenges in TB control, especially in regions with dense populations, high levels of poverty, and limited access to healthcare. Understanding and addressing these challenges is crucial for global health, as TB not only affects individual health outcomes but also has significant socioeconomic impacts, particularly in low- and middle-income countries (LMICs) [3].

Since the early 1990s, global initiatives, including the United Nations Millennium Development Goals (UNMDS), the WETS, and the United Nations Sustainable Development Goals (UN-SDGs), have sought to reduce the TB burden worldwide [1]. These efforts have led to some success, with global TB mortality declining by nearly 47 % between 1990 and 2015 [4]. However, progress has been uneven, particularly in South Asia, where the high TB burden persists despite these global efforts [4]. The WETS targets a 95 % reduction in TB deaths and a 90 % decrease in TB incidence by 2035. It set ambitious goals for 2020, including a 35 % decrease in TB deaths and a 20 % reduction in TB incidence [5]. However, according to the 2022 Global TB Report, TB deaths decreased by only 5.9 %, and the TB incidence rate decreased by just 10 % between 2015 and 2021, which is far below the targeted milestones [6]. Moreover, the global decline in TB incidence has stagnated, with annual rates of decline hovering between 1 % and 2 % over the past decade [7]. These statistics underscore the need for a more focused and region-specific approach to TB control in South Asia, where the disease burden remains disproportionately high.

The UN-SDGs and the WETS have set ambitious targets to significantly reduce the incidence of TB by 2030 [8], [9]. These global initiatives aim not only to decrease the number of TB cases but also to curb TB-related mortality, and they emphasize universal access to prevention, diagnosis, and treatment. Given the high burden of TB in South Asia, particularly in countries such as India, Pakistan, and Bangladesh, evaluating the effectiveness of ongoing efforts in this region is critical. This involves monitoring progress towards the set targets, identifying challenges, and making necessary adjustments to strategies. Additionally, it is important to project the future incidence of TB in South Asia to ensure that the region is on track to meet the 2030 targets. Such assessments will help policymakers and health organizations better allocate resources, implement targeted interventions, and ultimately achieve the goal of ending the TB epidemic in South Asia.

This study distinguishes itself by providing a comprehensive, three-decade regional analysis using GBD 2021 data to assess trends in ASIR, ASPR, ASMR, and DALYs related to TB. By employing advanced statistical models such as joinpoint regression and ARIMA forecasting, this work offers not only an in-depth historical review of TB trends from 1990 to 2021 but also projections up to 2031, which are rare in existing literature. Moreover, this study delves into the role of key risk factors like tobacco use, high fasting plasma glucose, and high body mass index, providing a nuanced understanding of how these factors differentially impact TB burden across South Asian countries. This study aims to inform targeted interventions and strategies that can accelerate progress towards the WETS targets, ultimately contributing to the global fight against TB.

## 2 Methods

### 2.1 Data source

The data source for the GBD 2021 study was overseen by the Institute for Health Metrics and Evaluation (IHME). The GBD 2021 study assesses the impact of diseases, injuries, and risk factors across 204 countries and subnational regions [10]. For this study, data were downloaded for ASIR, ASPR, ASMR, and ASDR of TB in South Asian countries from 1990 to 2021 (https://vizhub.healthdata.org/gbd-results/). Additionally, data on DALYs attributable to risk factors among TB patients in South Asia for 2021 were also obtained. In GBD 2021, data are provided for South Asian countries, including Bangladesh, Bhutan, India, Nepal, and Pakistan.

### 2.2 Statistical analysis

This study employed joinpoint regression analysis to rigorously examine the trends in ASIR, ASPR, ASMR, and ASDR related to TB in South Asia [11]. This analysis was conducted via the Joinpoint Regression Program (Version 5.2.0, National Cancer Institute) where the automated algorithm identifies and selects key points in the time trend series [12]. The joinpoint regression method employs a permutation test with an overall significance level set at 0.05 to assess the statistical significance of these changes. Additionally, the analysis utilized a constant variance method for correlated errors, ensuring that the variance remained consistent across the data points, thereby providing a more robust assessment of the trends. In addition to identifying historical trends, this study also aimed to predict future TB incidence rates. To achieve this goal, the ARIMA (autoregressive integrated moving average) model was applied via the “Forecast” package in R software [13]. ARIMA is a widely used time series forecasting model that is particularly effective in capturing the underlying patterns in data and predicting future values [14]. By inputting historical ASIR data into the ARIMA model, the TB incidence rates in South Asia were projected to 2031. To project future TB incidence in South Asia, a model was selected based on three parameters: p (autoregressive order), d (degree of differencing), and q (moving average order). We chose an ARIMA (0,2,2) model based on the characteristics of historical TB incidence data from 1990 to 2021. A second-order differencing (d = 2) was necessary to achieve stationarity, addressing the strong trend in the data. The autoregressive term (p = 0) was set to zero due to the absence of significant autocorrelation among lagged values, while the moving average term (q = 2) was included to account for residual patterns and correct forecast errors from the last two periods. The Akaike Information Criterion (AIC) and Bayesian Information Criterion (BIC) were used to identify the best-fitting ARIMA models. Similarly, country-wise projections were also conducted using the same methodology. Using QGIS software (version 3.38.0), boundary maps of South Asia were generated to visualize the ASPR, ASMR, and their annual percentage changes (APCs) from 1990 to 2021 [15]. These maps were used to compare both the current values and the changes in these metrics over time across the region. A heatmap based on the DALYs attributable to risk factors among TB patients was created via MS Excel to analyse the most significant risk factors affecting the DALYs of TB patients in the region.

## 3 Results

**Fig. 1:**
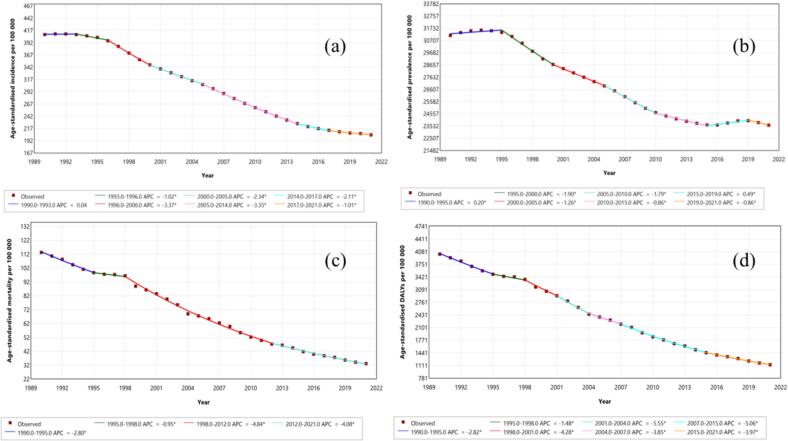
Join point analysis on age-standardised rates of Tuberculosis South Asia from 1990 to 2021. (a): Incidence per 100000; (b): Prevalence per 100000; (c): Mortality per 100000; (d): Disability-adjusted life years per 100000.

The joinpoint analysis of age-standardized rates of TB in South Asia from 1990 to 2021 revealed significant declining trends across incidence, prevalence, mortality, and DALY rates (Fig. 1). The incidence rate per 100,000 people showed notable decreases from 1996 to 2000, with a value of −3.37 (95 % CI: −3.22 to −3.63), and from 2005 to 2014, with a value of −2.11 (95 % CI: −1.82 to −2.72), whereas the prevalence rate experienced a significant decline between 1995–2000, with a value of −1.9 (95 % CI: −1.79 to −2.09) (Table 1). ASMR also displayed a continuous significant reduction, particularly from 1998 to 2012, with −4.84 (95 % CI: −3.35 to −5.79), and DALYs demonstrated consistent decreases, most notably during the periods from 1998 to 2001 and 2015–2021, with −4.28 (−1.88 to −5.35) and −3.97 (−3.49 to −4.43) APC, respectively (Table 1). The average annual percentage change (AAPC) from 1990 to 2021 showed a declining trend in all four metrics, including the ASIR (−2.22, 95 % CI: −2.2 to −2.24), ASPR (−0.9, 95 % CI: −0.88 to −0.92), ASMR (−4, 95 % CI: −3.96 to −4.05), and ASDR (−3.92, 95 % CI: −3.82 to −4.02). Overall, these trends indicate substantial progress in reducing the TB burden in South Asia over the three decades.

**Table 1:** Joinpoint analysis results showing the annual percentage change in age-standardised rates for incidence, prevalence, DALYs and mortality of TB in South Asia from 1990 to 2021.

	Age-standardised incidence rate		Age-standardised prevalence rate		Age-standardised DALYs^$^ rate		Age-standardised mortality rate	
Segment	**Year**	**APC^$^** **(95 % C.I^$^)**	**Year**	**APC** **(95 % C.I)**	**Year**	**APC** **(95 % C.I)**	**Year**	**APC** **(95 % C.I)**
1	1990 to 1993	0.04 (0.34 to −0.13)	1990 to 1995	0.2* (0.31 to 0.1)	1990 to 1995	−2.82* (−2.48 to −3.51)	1990 to 1995	−2.8* (−1.92 to −4.63)
2	1993 to 1996	−1.02* (−0.79 to −1.22)	1995 to 2000	−1.9* (−1.79 to −2.09)	1995 to 1998	−1.48* (−1.03 to −2.71)	1995 to 1998	−0.95* (−0.11 to −5.25)
3	1996 to 2000	−3.37* (−3.22 to −3.63)	2000 to 2005	−1.26* (−0.99 to −1.39)	1998 to 2001	−4.28* (−1.88 to −5.35)	1998 to 2012	−4.84* (−3.35 to −5.79)
4	2000 to 2005	−2.34* (−2.15 to −2.49)	2005 to 2010	−1.79* (−1.67 to −2.06)	2001 to 2004	−5.55* (−3.83 to −5.99)	2012 to 2021	−4.08* (−3 to −5.23)
5	2005 to 2014	−3.35* (−3.3 to −3.41)	2010 to 2015	−0.86* (−0.67 to −1)	2004 to 2007	−3.85* (−3.46 to −5.44)		
6	2014 to 2017	−2.11* (−1.82 to −2.72)	2015 to 2019	0.49* (0.71 to 0.35)	2007 to 2015	−5.06* (−3.71 to −5.45)		
7	2017 to 2021	−1.01* (−0.72 to −1.16)	2019 to 2021	−0.86* (−0.4 to −1.21)	2015 to 2021	−3.97* (−3.49 to −4.43)		
AAPC^$^	1990–2021	−2.22* (−2.2 to −2.24)	1990–2021	−0.9* (−0.88 to −0.92)	1990–2021	−3.92* (−3.82 to −4.02)	1990–2021	−4* (−3.96 to −4.05)

^$^AAPC: Average annual percentage change; APC: Annual percentage change; DALYs: Disability-adjusted life years; C.I: Confidence intervals

*Indicates that APC and AAPC is significantly different from zero at the alpha = 0.05 level.

Note: The Annual Percent Change (APC) is a method used to describe changes in disease rates over time.

**Fig. 2:**
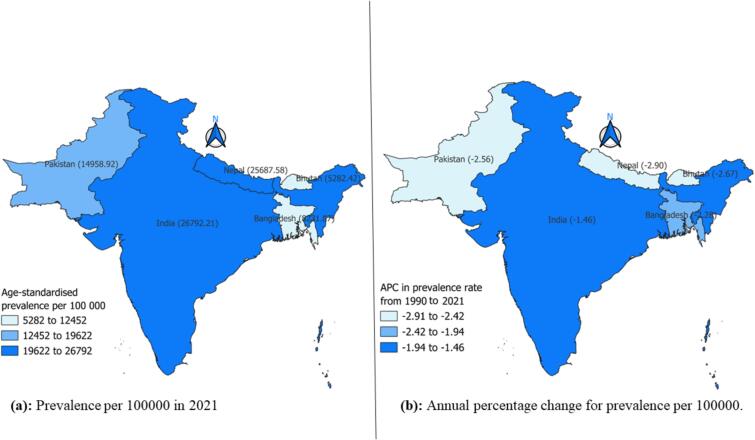
Boundary map of South Asia showing the age-standardised prevalence rate and the annual percentage change in the prevalence rate of TB from 1990 to 2021.

Fig. 2 shows the ASPR of TB and APC from 1990 to 2021 in South Asia. The map illustrates that the highest ASPR was observed in India, with 26,792.21 cases per 100,000, followed by Nepal and Pakistan, with ASPR of 25,687.58 and 14,958.92, respectively (Fig. 2a). Although TB incidence is declining overall, among South Asian countries, India has shown the smallest decline, with an APC of −1.46 in the ASPR from 1990 to 2021 (Fig. 2b).

**Fig. 3:**
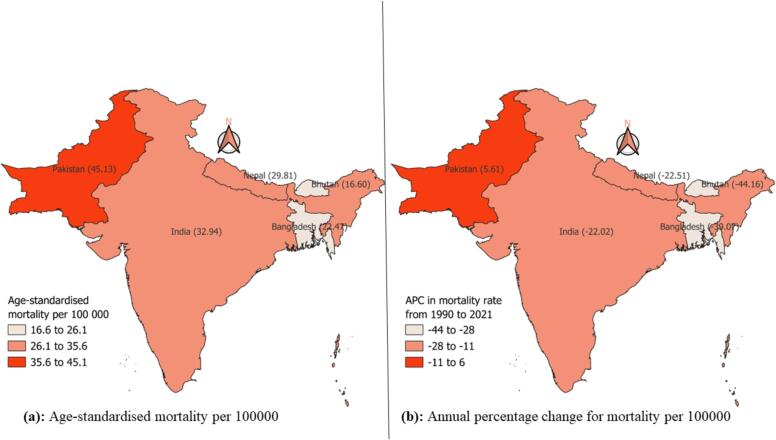
Boundary map of South Asia showing the age-standardised mortality rate and the annual percentage change in the mortality rate of TB from 1990 to 2021.

In 2021, Pakistan had the highest ASMR of 45.13 deaths per 100,000, followed by India, Nepal, and Bangladesh, with ASMR of 32.94, 29.81, and 22.47, respectively (Fig. 3a). Pakistan showed an increase in the ASMR from 1990 to 2021, with an APC of 5.61 %. In contrast, declining results were observed among other countries, including India and Nepal, Bangladesh and Bhutan, with values of –22.02, –22.51, −39.07 and −44.16 APC, respectively (Fig. 3b).

Overall, the region experienced a significant reduction in both TB ASIR and ASDR, with South Asia showing APCs of −0.5 (95 % UI: −0.46 to −0.54) and −0.72 (95 % UI: −0.64 to −0.76), respectively. Bangladesh and Bhutan exhibited the most substantial declines, with Bangladesh's ASIR and ASDR decreasing by −0.73 (95 % UI: −0.7 to −0.76) and −0.85 (95 % UI: −0.79 to −0.89), respectively, and Bhutan's ASIR and ASDR decreasing by −0.59 (95 % UI: −0.55 to −0.64) and −0.81 (95 % UI: −0.72 to −0.87), respectively. India also recorded a reduction in ASIR and ASDR of −0.45 (95 % UI: −0.39 to −0.51) and −0.71 (95 % UI: −0.63 to −0.76), respectively. Similarly, Nepal and Pakistan showed decreases, with Nepal showing a −0.67 (95 % UI: −0.63 to −0.7) reduction in ASIR and a −0.82 (95 % UI: −0.71 to −0.88) reduction in ASDR, whereas Pakistan’s ASIR decreased by −0.56 (95 % UI: −0.52 to −0.59) and the ASDR decreased by −0.58 (95 % UI: −0.45 to −0.7). These results highlight a consistent downwards trend in the TB burden across the region over the study period (Table 2).

**Table 2:** Country wise annual percentage change in age-standardised rates for incidence and DALYs of TB from 1990 to 2021 in South Asia.

	1990 age-standardised rates		2021 age-standardised rates		APC^$^ from 1990 to 2021	
States	**Incidence (U.I^$^)**	**DALYs^$^(U.I)**	**Incidence (U.I)**	**DALYs (U.I)**	**Incidence (U.I)**	**DALYs (U.I)**
South Asia	408.7 (488.64 to 342.56)	4018.2 (4459.32 to 3497.82)	204.05 (231.66 to 180.62)	1133.21 (1304.29 to 1015.74)	−0.5 (−0.46 to −0.54)	−0.72 (−0.64 to −0.76)
Bangladesh	524.87 (598.86 to 458.44)	4765.31 (5594 to 3802.25)	140.67 (161.94 to 123.1)	697.67 (866.41 to 558.81)	−0.73 (−0.7 to −0.76)	−0.85 (−0.79 to −0.89)
Bhutan	279.53 (323.96 to 239.99)	2750.24 (4750.14 to 1475.25)	113.36 (131.25 to 97.8)	521.74 (943.18 to 284.62)	−0.59 (−0.55 to −0.64)	−0.81 (−0.72 to −0.87)
India	392.62 (477.11 to 323.9)	3910.27 (4366.49 to 3490.87)	214.39 (244.86 to 188.98)	1119.71 (1355.66 to 977.01)	−0.45 (−0.39 to −0.51)	−0.71 (−0.63 to −0.76)
Nepal	475.77 (517.19 to 432.96)	4967.72 (6380.23 to 3697.92)	157.43 (179.79 to 137.16)	911.35 (1219.2 to 652.41)	−0.67 (−0.63 to −0.7)	−0.82 (−0.71 to −0.88)
Pakistan	415.94 (474.79 to 364.4)	3736.09 (5160.27 to 2391.38)	182.83 (208.49 to 161.59)	1565.3 (1953.48 to 1160.47)	−0.56 (−0.52 to −0.59)	−0.58 (−0.45 to −0.7)

**^$^**Indicates: APC: Annual percentage change; DALYs: Disability-adjusted life years, Uncertainty interval.

Note: The Annual Percent Change (APC) is a method used to describe changes in disease rates over time.

**Fig. 4:**
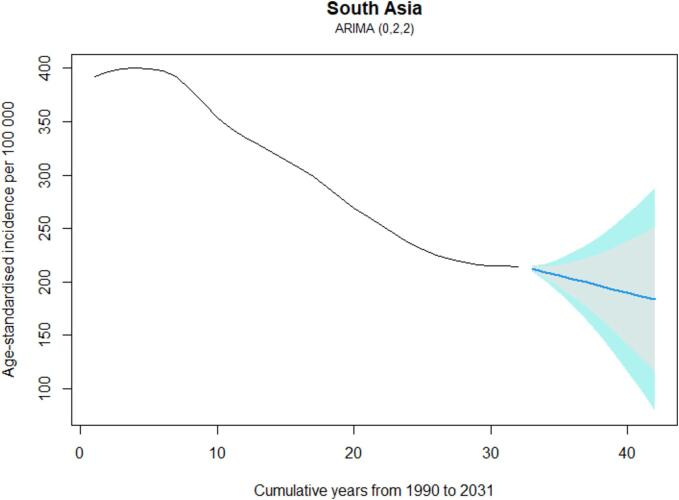
Projection of TB from 1990 to 2031 in South Asia using Auto-regressive integrated moving average (ARIMA) model (0, 2, 2). Note: Shaded part indicating the 95% and 80% uncertainty intervals.

Fig. 4 projects the trends in TB incidence in South Asia from 1990 to 2031 via the ARIMA (0 2 2) model. The projections indicate a declining trend in TB incidence across South Asia, with the most significant reductions observed in the later years of the projection period. Table 3 presents the projected ASIR of TB in South Asia and specific countries (India, Bangladesh, Bhutan, Nepal, and Pakistan) from 2022 to 2031. The projections indicate a general decline in the TB ASIR across all regions, with South Asia as a whole showing a steady decrease from 200.83 in 2022 to 171.64 ASIR in 2031. The TB incidence rate in India is projected to decrease from 211.04 ASIR in 2022 to 180.96 ASIR in 2031. Bangladesh shows a significant decline from 129.99 ASIR in 2022 to 33.89 ASIR in 2031. Similarly, Bhutan's TB incidence is expected to decrease from 109.27 in 2022 to 72.39 ASIR in 2031. Nepal is projected to initially increase in incidence rates, peaking at 207.59 per 100,000 in 2031. Pakistan's projections show a dramatic decline from 168.02 ASIR in 2022 to just 20.62 per 100,000 in 2031, with considerable uncertainty in the later years. Overall, the projections suggest an encouraging trend of declining TB incidence in the region, although the extent of decline varies by country.

**Table 3:** Incidence forecasting of TB disease in South Asia from 2022 to 2031 based on GBD data from 1990 to 2021.

Year	South Asia ARIMA^$^ (0,2,2)	India ARIMA (0,2,1)	Bangladesh ARIMA (0,2,1)	Bhutan ARIMA (0,2,1)	Nepal ARIMA (1,2,2)	Pakistan ARIMA (0,2,2)
2022	200.83 (146.91 to 254.75)	211.04 (208.45 to 213.64)	129.99 (127.19 to 132.79)	109.27 (108.41 to 110.12)	157.09 (155.54 to 158.64)	168.02 (166.4 to 169.64)
2023	197.59 (118.22 to 276.96)	207.7 (199.73 to 215.67)	119.31 (111.73 to 126.89)	105.17 (102.81 to 107.53)	160.93 (156.42 to 165.43)	151.64 (145.89 to 157.39)
2024	194.34 (95.38 to 293.31)	204.36 (189.54 to 219.18)	108.63 (95.04 to 122.23)	101.07 (96.81 to 105.33)	166.18 (156.35 to 176.01)	135.26 (123.55 to 146.97)
2025	191.1 (75.37 to 306.83)	201.02 (178.13 to 223.9)	97.96 (77.31 to 118.6)	96.97 (90.49 to 103.45)	171.9 (154.9 to 188.91)	118.89 (99.94 to 137.83)
2026	187.86 (57.12 to 318.6)	197.67 (165.66 to 229.69)	87.28 (58.69 to 115.87)	92.87 (83.88 to 101.87)	177.79 (152.14 to 203.44)	102.51 (75.27 to 129.75)
2027	184.61 (40.06 to 329.17)	194.33 (152.24 to 236.43)	76.6 (39.25 to 113.95)	88.78 (77.02 to 100.53)	183.73 (148.18 to 219.28)	86.13 (49.65 to 122.61)
2028	181.37 (23.88 to 338.86)	190.99 (137.94 to 244.04)	65.92 (19.08 to 112.76)	84.68 (69.93 to 99.43)	189.69 (143.15 to 236.23)	69.75 (23.18 to 116.33)
2029	178.13 (8.38 to 347.87)	187.65 (122.84 to 252.46)	55.24 (−1.78 to 112.27)	80.58 (62.61 to 98.55)	195.65 (137.13 to 254.18)	53.37 (−4.08 to 110.83)
2030	174.88 (−6.57 to 356.33)	184.31 (106.98 to 261.63)	44.57 (–23.28 to 112.41)	76.48 (55.09 to 97.87)	201.62 (130.21 to 273.03)	37 (–32.07 to 106.06)
2031	171.64 (−21.08 to 364.35)	180.96 (90.41 to 271.52)	33.89 (−45.39 to 113.17)	72.39 (47.38 to 97.39)	207.59 (122.44 to 292.73)	20.62 (−60.75 to 101.99)

^$^ARIMA (Autoregressive Integrated Moving Average) is a statistical method used to forecast future disease trends based on historical data.

**Fig. 5:**
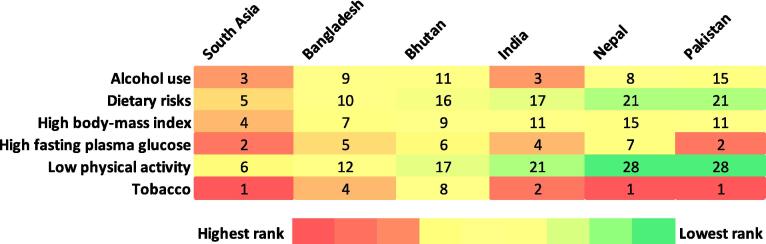
Heatmap analysis showing ranking of South Asian countries for DALYs attributable to risk factors among TB patients in 2021.

Fig. 5 shows a heatmap analysis showing the ranking of South Asian countries for DALYs attributable to various risk factors among TB patients in 2021. Tobacco use ranks as the highest contributing risk factor across all countries, with Pakistan, India, and Bangladesh holding the top three positions. High fasting plasma glucose and high body mass index have also emerged as significant contributors, particularly in Bangladesh, Bhutan, and India. Other factors, including dietary risk, low physical activity, and alcohol use, have varying levels of impact across countries, with dietary risk and low physical activity ranking lower in most countries.

## 4 Discussion

The aim of this study was to evaluate the trends in TB burden across South Asia from 1990 to 2021 and to project future TB incidence rates via the GBD framework. The major findings revealed significant declines in all health metrics, including the ASIR, ASPR, ASMR, and DALYs related to TB, across the region over the past three decades. These results suggest substantial progress in TB control efforts in South Asia, indicating that interventions implemented during this period positively impacted the TB burden. The study also developed projections for TB incidence up to 2031, highlighting a continued downwards trend across most countries, which could significantly contribute to achieving the targets set by the WETS.

The observed declining trends in ASIR, ASPR, ASMR, and DALYs in South Asia align with previous global studies that reported reductions in TB incidence and mortality due to enhanced public health interventions, including the expansion of TB control programs and the integration of TB services with broader healthcare systems [16], [17], [18]. For example, the consistent reduction in the ASMR from 1998 to 2012 correlated with intensified TB control efforts and the scaling-up of DOTS (Directly Observed Treatment, Short-course) in the region [19], [20], [21], [22]. However, the relatively smaller decline in TB incidence in India than in other South Asian countries highlights the persistent challenges in addressing the high TB burden. These challenges are exacerbated by densely populated areas, significant socioeconomic disparities, widespread malnutrition, and high rates of tobacco consumption [23], [24], [25].

Bangladesh and Bhutan have shown substantial declines in TB incidence and mortality rates, and other countries, such as Pakistan, have exhibited less consistent progress, with Pakistan even showing an increase in the ASMR from 1990 to 2021. This suggests that while some countries have successfully implemented and scaled up effective TB control measures, others continue to face significant challenges, potentially due to differences in healthcare infrastructure, political commitment, and socioeconomic factors [18], [26], [27], [28].

Moreover, the projections for TB incidence up to 2031, while generally positive, suggest that without continued and possibly intensified efforts, the region may still fall short of the WETS targets. Countries such as Nepal and Pakistan, which are projected to experience a rise or less significant decline in TB incidence, indicate that TB control efforts may be insufficient in addressing the underlying social determinants of health, such as poverty, malnutrition, healthcare access and insufficient awareness of the disease's symptoms and its potential for transmission in these countries [16], [29]. These social determinants are critical in TB control, as evidenced by the strong correlation between high DALYs attributable to risk factors such as tobacco use and high fasting plasma glucose [30], [31]. It was estimated that as many as 15,000 TB cases in Nepal could be associated with tobacco use [6]. Nepal's 2019 National TB Prevalence Survey estimates that 117,000 people in the country are living with TB, despite efforts that have resulted in a 3 % annual reduction in new TB cases over the past decade [32]. However, Nepal targets ending the TB epidemic by 2035. To evaluate progress, the National Tuberculosis Control Center (NTCC), with the WHO's support, carried out a TB Programme Review Mission in 2023 to identify programmatic gaps and challenges and strengthen the TB management system [17]. These risk factors need to be addressed. Tobacco use, which ranks as the highest risk across all South Asian countries, calls for stronger tobacco control policies. This could include implementing stricter regulations on tobacco sales, higher taxes, public awareness campaigns about the dangers of smoking, and integrating smoking cessation programs into TB care. Similarly, high fasting plasma glucose, a significant contributor to the TB burden, could be addressed through policies promoting better nutrition, regular health screenings for early detection of diabetes, and integrating diabetes management into TB control programs. Such targeted interventions would not only address these risk factors but also help accelerate the progress toward TB elimination in the region.

Despite the robust analysis, this study has several limitations that could have affected the results. First, While the ARIMA model provides valuable projections for future TB trends, it is important to recognize its limitations. The model is based on historical data and assumes that past trends will continue into the future, which may not hold true in the face of unexpected disruptions. Events such as pandemics (e.g., COVID-19), shifts in health policies, and socioeconomic changes can significantly impact TB detection, treatment, and incidence. The reliance on GBD 2021 data in this study provides a robust foundation for analysing TB trends; however, it may not fully account for the recent shifts in health policies or the long-term effects of the COVID-19 pandemic [33]. The pandemic disrupted healthcare systems globally, potentially altering TB detection, treatment, and control measures. Moreover, many countries, particularly in South Asia, implemented new health policies in response to the pandemic, which could have affected TB trends [34]. These factors warrant a more in-depth discussion, as they might have introduced fluctuations in TB incidence and mortality that the GBD 2021 data has not yet captured. Moreover, joinpoint regression analysis, while effective for identifying trends, might not fully capture the complexities of TB dynamics in different subregions or population groups within South Asia.

Future research should focus on continuously updating TB burden assessments to incorporate new data and emerging trends, particularly in the post pandemic context, where disruptions in healthcare services might have impacted TB control. Additionally, there is a need for more localized studies that explore TB trends and control efforts within specific subregions or vulnerable populations in South Asia to tailor interventions more effectively. Expanding research on the social determinants of TB, such as poverty and malnutrition, could also provide insights into addressing the root causes of TB transmission and persistence. Finally, integrating more advanced predictive models that consider a broader range of factors, including policy shifts and healthcare innovations, could enhance the accuracy of future projections.

## 5 Conclusions

In conclusion, this study provides critical insights into the trends and future projections of the TB burden in South Asia, highlighting significant progress in reducing TB incidence and mortality over the past three decades. These findings emphasize the importance of sustained and targeted efforts in TB control to maintain and accelerate this progress towards the WETS. The study's contribution lies in its comprehensive regional analysis, which not only informs policymakers of the current state of TB in South Asia but also guides future strategies to ensure that the region remains on track to meet global TB elimination targets. The relevance and significance of these findings are paramount, as South Asia's success in TB control will be crucial for achieving global health milestones.

## Approval of the research protocol by an Institutional Reviewer Board

Not applicable.

## Registry and the Registration No. of the study/trial

Not applicable.

## Animal studies

Not applicable.

## Availability of data and materials

Available at Institute for Health Metrics and Evaluation (IHME) site (https://vizhub.healthdata.org/gbd-results/).

## Ethics, consent to participate, and consent to publish declarations

Not applicable.

## Informed consent

Not applicable.

## Funding

No funding.

## CRediT authorship contribution statement

**Vijay Kumar:** Writing – review & editing, Writing – original draft, Visualization, Software, Methodology, Formal analysis, Data curation, Conceptualization. **Mahalaqua Nazli Khatib:** Writing – review & editing, Software, Methodology, Formal analysis, Conceptualization. **Amit Verma:** Writing – review & editing, Visualization, Methodology. **Sorabh Lakhanpal:** Writing – review & editing, Writing – original draft, Conceptualization. **Suhas Ballal:** Writing – original draft, Validation, Software. **Sanjay Kumar:** Writing – review & editing, Writing – original draft. **Mahakshit Bhat:** Visualization, Formal analysis, Conceptualization. **Aryantika Sharma:** Writing – review & editing, Writing – original draft. **M. Ravi Kumar:** Writing – review & editing, Validation. **Aashna Sinha:** Writing – original draft, Visualization, Software. **Abhay M. Gaidhane:** Writing – original draft, Methodology. **Muhammed Shabil:** Writing – original draft, Conceptualization. **Mahendra Pratap Singh:** Writing – original draft, Methodology. **Sanjit Sah:** Writing – original draft, Methodology, Conceptualization. **Kiran Bhopte:** Software, Writing – original draft. **Kamal Kundra:** Methodology, Writing – original draft. **Shailesh Kumar Samal:** Conceptualization, Methodology, Writing – review & editing.

## Declaration of competing interest

The authors declare that they have no known competing financial interests or personal relationships that could have appeared to influence the work reported in this paper.
